# Diacylglycerol Kinases Are Widespread in Higher Plants and Display Inducible Gene Expression in Response to Beneficial Elements, Metal, and Metalloid Ions

**DOI:** 10.3389/fpls.2017.00129

**Published:** 2017-02-07

**Authors:** Hugo F. Escobar-Sepúlveda, Libia I. Trejo-Téllez, Paulino Pérez-Rodríguez, Juan V. Hidalgo-Contreras, Fernando C. Gómez-Merino

**Affiliations:** ^1^Colegio de Postgraduados Campus Córdoba, Amatlán de los ReyesVeracruz, Mexico; ^2^Colegio de Postgraduados Campus MontecilloTexcoco, Mexico

**Keywords:** phospholipids, glycerol, plant signaling, hormesis, beneficial elements, Genevestigator

## Abstract

Diacylglycerol kinases (DGKs) are pivotal signaling enzymes that phosphorylate diacylglycerol (DAG) to yield phosphatidic acid (PA). The biosynthesis of PA from phospholipase D (PLD) and the coupled phospholipase C (PLC)/DGK route is a crucial signaling process in eukaryotic cells. Next to PLD, the PLC/DGK pathway is the second most important generator of PA in response to biotic and abiotic stresses. In eukaryotic cells, DGK, DAG, and PA are implicated in vital processes such as growth, development, and responses to environmental cues. A plethora of DGK isoforms have been identified so far, making this a rather large family of enzymes in plants. Herein we performed a comprehensive phylogenetic analysis of DGK isoforms in model and crop plants in order to gain insight into the evolution of higher plant DGKs. Furthermore, we explored the expression profiling data available in public data bases concerning the regulation of plant *DGK* genes in response to beneficial elements and other metal and metalloid ions, including silver (Ag), aluminum (Al), arsenic (As), cadmium (Cd), chromium (Cr), mercury (Hg), and sodium (Na). In all plant genomes explored, we were able to find DGK representatives, though in different numbers. The phylogenetic analysis revealed that these enzymes fall into three major clusters, whose distribution depends on the composition of structural domains. The catalytic domain conserves the consensus sequence GXGXXG/A where ATP binds. The expression profiling data demonstrated that *DGK* genes are rapidly but transiently regulated in response to certain concentrations and time exposures of beneficial elements and other ions in different plant tissues analyzed, suggesting that DGKs may mediate signals triggered by these elements. Though this evidence is conclusive, further signaling cascades that such elements may stimulate during hormesis, involving the phosphoinositide signaling pathway and DGK genes and enzymes, remain to be elucidated.

## Introduction

Plants have the remarkable capability of responding to environmental cues thanks to different signal transduction cascades, which are triggered by signaling molecules that perceive and expand external and internal signals, resulting in plant adaptation reactions (Arisz et al., 2009; Sparks et al., 2013; Abd-El-Haliem et al., 2016). Some of the most important signaling molecules in plants involve lipids, including phosphoinositides (PPI), sphingolipids, lysophospholipids, oxylipins, *N*-acylethanolamines, and free fatty acids (Wang and Chapman, 2013). Though most of the leaf structural lipids in plant cells are galactolipids (approximately 70%), phospholipids, including phosphatidic acid (PA), play important roles in signal perception and transduction (Testerink and Munnik, 2005).

PA has been proposed to be a pivotal second messenger in plants and its synthesis has been reported to be induced in response to ethylene (Munnik, 2001), abscisic acid (Zhang et al., 2004), wounding and Nod factor (Munnik, 2001), osmotic pressure (Munnik et al., 2000; Testerink et al., 2004), cold (Ruelland et al., 2002), salinity (Zhang et al., 2012), temperature changes (Arisz et al., 2013), pathogen attack (Zhang and Xiao, 2015), and drought (Li et al., 2015). In all of those cases, PA synthesis has been associated with plant cell adjustments to overcome such stress events. Nevertheless, some positive-strand RNA viruses use PA in order to stimulate their replication (Hyodo et al., 2015), which has to be taken into consideration when designing strategies to apply PA as a potential biostimulator of adaptive responses in crop plants. Importantly, PA is a precursor to all phosphoglycerolipids as well as triacylglycerols and galactolipids, and its turnover is crucial in determining lipid metabolic fluxes and membrane compositions (Arisz et al., 2009, 2013).

In plant cells, PA can be generated in the plasma membrane from two different metabolic pathways: (1) as a product of the hydrolysis of structural phospholipids, such as phosphatidylcholine and phosphatidylethanolamine by the action of different isoforms of phospholipase D (PLD; Hong et al., 2014); or (2) through the combined activity of phospholipase C (PLC) and diacylglycerol kinase (DGK). There are two types of PLCs in plant cells: those that take PPI as substrate, the so called phosphatidylinositol (PI)-PLCs, and those that hydrolyze structural phospholipids, known as non-specific PLCs (NPCs). In any case, PLCs yield diacylglycerol (DAG) and inositol 1,4,5-trisphosphate (IP_3_). After phosphatidylinositol 4,5-bisphosphate hydrolysis, IP_3_ diffuses into the cytosol and acts as a second messenger implicated in calcium (Ca^2+^) mobilization from intracellular compartments such as the vacuole, while DAG remains in the plasma membrane and may activate other physiological processes. DAG can then be phosphorylated by DGK to yield PA, which in turn can be further metabolized to PPI. Additionally, enzymes that dephosphorylate PA include lipid phosphate phosphatases and PA hydrolases (lipins). Moreover, PA can be further phosphorylated to diacylglycerol pyrophosphate by PA kinase, or metabolized to lyso-PA through PLA_2_ activity (Testerink and Munnik, 2011). Most of these lipids and enzymes have been proved to have signaling functions, and the synthesis of signaling lipids in response to different environmental cues and stressors is essentially transient (Testerink and Munnik, 2011; Ge et al., 2012).

In eukaryotic cells, PA levels are typically low, corresponding to 0.67% of total phospholipids (Arisz et al., 2000) and PA formation depends on the extracellular stimuli perceived.

In the green unicellular algae *Chlamydomonas moewusii*, hyperosmotic stress triggers the activation of both PA biosynthetic pathways (i.e., PLD and PLC/DGK; Munnik et al., 2000). Indeed, with the activation of mastoparan (a potent activator of PLC and PLD signaling in plants), 5–17% of PA is generated by PLD, while the rest is assumed to be generated by the PLC/DGK (Munnik et al., 1998). In cold-shock Arabidopsis stimulated cells (0°C), during the first 10 min up to 80% of PA is produced via the coordinated PLC/DGK pathway, whereas afterward the PLD pathway dominates (Ruelland et al., 2002). In Arabidopsis, the induction of programmed cell death due to the accumulation of H_2_O_2_ caused by UV irradiation or drought-induced dehydration is drastically reduced as a consequence of an overproduction of PA via PLD (Zhang et al., 2003). Interestingly, oxidative stress and wounding boost PA synthesis via the PLD pathway too (Munnik, 2001; Testerink and Munnik, 2005). This pathway (PLD) is also responsible for the total production of PA in Arabidopsis cells exposed to pathogens (de Torres Zabela et al., 2002). Nevertheless, in tomato cells exposed to the elicitors CH4 and flg22, PA biosynthesis is stimulated by PLC/DGK, whereas PLD does not show activity (van der Luit et al., 2000).

**FIGURE 1:**
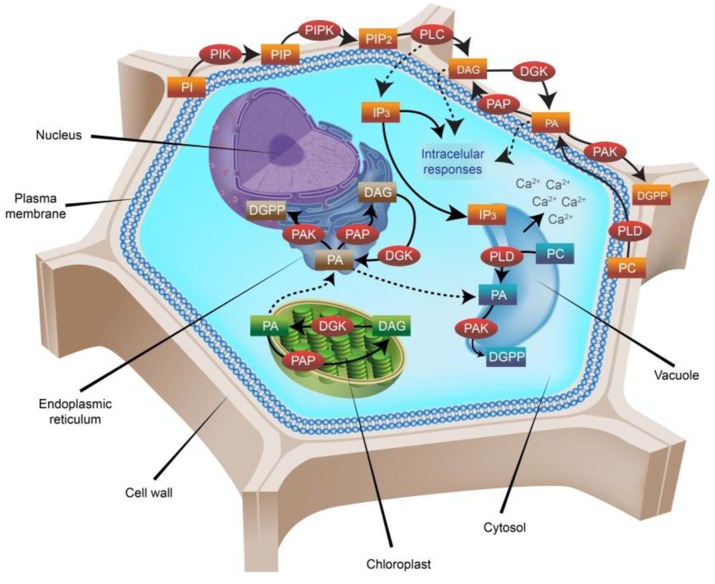
**Phosphatidic acid (PA) metabolic pathways in higher plants**. Nearly 30% of the plasma membrane in plants is composed of phospholipids, which can form lipid bilayers because of their amphiphilic properties. All phospholipids based on phosphatidylinositol (PI) are referred to as phosphoinositides (PPI). PIs are formed by two fatty-acid chains bounded to an inositol ring through a phosphodiester linkage. Phosphorylations of the inositol ring are carried out by specific PI kinases (PIK), including phosphatidylinositol 3-kinase (PI3K), phosphatidylinositol 4-kinase (PI4K) and phosphatidylinositol 5-kinase (PI5K) at the D-3, D-4, or D-5 positions, yielding phosphatidylinositol 3-phosphate (PI3P), phosphatidylinositol 4-phosphate (PI4P) or phosphatidylinositol 5-phosphate (PI5P), respectively. Sequential phosphorylation by phosphatidylinositol 5-phosphate 4-kinase (PIP4K) or phosphatidylinositol 4-phosphate 5-kinase (PIP5K) forms phosphatidylinositol 4,5-bisphosphate (PIP2), which is then hydrolyzed by phospholipase C (PLC), resulting in the production of two important second messengers: inositol trisphosphate (IP_3_) and diacylglycerol (DAG). The hydrophilic IP_3_ diffuses into the cytosol where it triggers Ca^2+^ release from intracellular stores, whereas the lipophilic DAG molecule remains in the membrane and participates in other signaling processes. DAG can be further phosphorylated by the enzyme diacylglycerol kinase (DGK), giving rise to phosphatidic acid (PA). Additionally, PLD hydrolyses phospholipids at the terminal phosphodiester bond and generates PA too. Further phosphorylation of PA by the enzyme PA kinase (PAK) generates diacylglycerol pyrophosphate (DGPP), which is also a signaling molecule in plant cells. Dephosphorylation of PA by the enzyme PA phosphatase (PAP) generates DAG again. DGK enzymes are present not only in the plasmalemma, but also in the endoplasmic reticulum (ER) and the chloroplast membranes. The PA produced in the chloroplast is transported to the ER, where it renders either DGPP (by a further phosphorylation carried out by PAK) or DAG (by the activity of PAP). In plasma membranes, the most abundant PPI is PIP2, where it comprises ∼1% of the total phospholipid pool present. Importantly, not all PPI signaling molecules are involved in signal perception and transduction all the time. Indeed, most of them are constantly participating in the biosynthesis and turnover of other phospholipids, and are recruited for signaling proposes just when needed. PA itself is not always expressed, and the magnitude of its variations looks crucial for plants. In the diagram, enzymes are represented in red circles, whereas substrates and products in rectangles are colored according to the localization of their origin: brown, ER; blue, vacuole; green, chloroplast; orange, plasma membrane.

Based on significant contributions by a number of plant biologists (Munnik et al., 1998; Munnik, 2001; Gómez-Merino et al., 2004; Cai et al., 2009; Kirik and Mudgett, 2009; Xue et al., 2009; Gonorazky et al., 2010; Arisz and Munnik, 2011; Dubots et al., 2011; Shin and Loewen, 2011; Boss and Im, 2012; Arisz et al., 2013; Villasuso et al., 2013; Logothetis et al., 2015; Saucedo-García et al., 2015; Singh et al., 2015; Hou et al., 2016), **Figure 1** shows the main metabolic pathways involving PLD, PLC, and DGK in the biosynthesis and turnover of PA in plant cells.

Eukaryotic DGKs are a widespread family of enzymes, well conserved among most multicellular organisms. Plant DGK isoforms have a conserved catalytic domain with an ATP-binding site (consensus GXGXXG/A) required for kinase activity (Gómez-Merino et al., 2004). DGK activity is likely to be tightly regulated to control DAG and PA levels coordinately, enabling the cell to perform its physiological functions appropriately (Gómez-Merino et al., 2004). In higher plants, DGK enzymes are present in different isoforms and their activity has been proved in tomato (*Solanum lycopersicum*; SlDGK; Snedden and Blumwald, 2000), Arabidopsis (AtDGK; Gómez-Merino et al., 2004, 2005a), maize (*Zea mays*; ZmDGK; Sui et al., 2008), wheat (*Triticum aestivum*; TaDGK; Zhang et al., 2008), rice (*Oryza sativa*; OsDGK; Ge et al., 2012), and apple (*Malus prunifolia*; MpDGK; Li et al., 2015), among others. To date, cumulative evidence points to a crucial role of DGK enzymes in vital processes like growth, development and responses to environmental stimuli and stresses. Phylogenetic analyses have been performed for some plant species, including Arabidopsis and rice DGKs, though a more robust evolution analysis of DGKs in higher plants is still lacking, and their implications in response to nutrients, including potential and well-known beneficial elements, await further studies. Herewith we describe the physiological roles of characterized DGK from higher plants isolated so far and present a comprehensive phylogenetic analysis of DGK isoforms identified in the proteomes of important model and cultivated plants. Furthermore, based on the Genevestigator platform (Zimmermann et al., 2014) and experimental data, we also report a detailed expression profiling analysis of *DGK* genes in response to known beneficial elements such as aluminum (Al) and sodium (Na), as well as other metal and metalloid ions, including silver (Ag), arsenic (As), cadmium (Cd), chromium (Cr), and mercury (Hg). Beneficial elements are not required by all plants, but may be essential for certain taxa depending on environmental conditions, element concentration and plant species. When applied at low concentrations, they may elicit positive responses in growth, yield, and responses to environmental stresses (Pilon-Smits et al., 2009; Poschenrieder et al., 2013). Indeed, beneficial elements can prompt hormesis, a stimulatory effect of a low dose of a non-essential element (Calabrese, 2014). Herewith we provide evidence for the first time that the expression of some *DGK* genes may be modified when plants are exposed to some of those hormesis-inducing elements. Furthermore, we explored the expression profiling data of such genes in different plant tissues.

## Materials and Methods

### Phylogenetic Analysis of DGKs in Higher Plants

To identify *DGK* gene homologs in plant species, searches were performed based on the reported sequences of *AtDGK* (Gómez-Merino et al., 2004) using the BLAST software^1^ (Altschul et al., 1990) with a Block Substitution Matrix 62 (BLOSUM62), considering the non-redundant sequences deposited in the National Center for Biotechnology Information (NCBI)^2^ and the UniProtKB server^3^. According to Henikoff and Henikoff (1992), this matrix is more appropriate for searches and alignments than are matrices derived by extrapolation from mutation rates. For the first analysis reported herein, results were filtered, selecting only the DGK sequences previously characterized or included in other phylogenetic studies and next extracted in FASTA format. Subsequently, we carried out a multiple sequence alignment of the Arabidopsis DGK protein collected in the previous stage with the Clustal-omega1.2.1 software (Sievers et al., 2011). This software performs a multiple sequence alignment using the Hidden Markov Model (HMM; Yoon, 2009), for later constructing a tree guide using Muscle fast unweighted pair group method with arithmetic mean (UPGMA) implementation (Edgar, 2004). We used three iterations of this algorithm to refine the alignment. Final alignment was then transformed to NXS format, which was used as entry to construct the final phylogenetic tree. This tree was built using an evolutive method implemented in the MrBayes3.2.5 software^4^ (Altekar et al., 2004). This method is robust, and it is based on Markov Chain Monte Carlo simulation methods. When constructing the phylogenetic tree we integrated 26 taxa considering the following analytical parameters: a priority matrix of fixed amino acids (aamodel = mixed); the evolutive WAG method with variations described using a gamma-type distribution (rates = invgamma) and default hyperparameters; three million generations (ngen = 3000000); frequency of tree sampling each 100 generations (samplefreq = 1000); another 750 samples of trees in the diagnostic phase (burnin = 750) were discarded. The rest of the trees were used to infer the further probabilities of the individual clades. In order to assess the convergence of the model, we ran several parallel chains, keeping the standard deviation from the result to a value below 0.01 (Ronquist et al., 2005). For that, we tested the software parameters, resulting in the following: number of generations: 3000000; temperature of chains: above 8°C.

### Expression Profiling Analyses of *DGK* Genes from Genevestigator

Tissue-specific expression patterns of available plant *DGK* gene probes were retrieved from the Genevestigator software package^5^ (Zimmermann et al., 2014). Furthermore, we were able to retrieve the expression profiling of Arabidopsis (*AtDGK*), rice (*OsDGK*), tomato (*SlDGK*), soybean (*GmDGK*), wheat (*TaDGK*) and barley (*HvDGK*) *DGK* genes in response to Ag, Al, As, Cd, Cr, Hg, and Na from Genevestigator as well.

## Results

### Phylogenetic Analysis of DGK Enzymes in Higher Plants

In order to reconstruct the DGK phylogeny from plants, a protein database search in the NCBI and UniProtKB was carried out. The queries were DGK sequences homolog to Arabidopsis DGKs using the BLAST tool. Subsequently, we added to our analysis all homolog DGK sequences previously analyzed phylogenetically and referred to in articles. From Arabidopsis, we took into consideration AtDGK1, AtDGK2, AtDGK3, AtDGK4, AtDGK5a, AtDGK5b, AtDGK6, and AtDGK7 (Gómez-Merino et al., 2004, 2005a); from rice, OsDGK1, OsDGK2, OsDGK3, OsDGK4, OsDGK5, OsDGK6, OsDGK7, and OsDGK8 (Ge et al., 2012); from maize, ZmDGK1, ZmDGK2, and ZmDGK3 (Sui et al., 2008); from wheat, TaDGK (Zhang et al., 2008); from tomato, SlDGK1a-b (Snedden and Blumwald, 2000); and from apple, MdGK1, MdDGK2, MdDGK5, and MdDGK7 (Li et al., 2015). Finally, each sequence was used as reference to perform the following searches of all DGK isoforms present in most crop plant proteomes, with a wider percentage of coverage.

After having completed the searches (47 DGK isoforms found in 22 plant species; **Table 1**), we aligned the sequences through Clustal-omega1.2.1 software (Sievers et al., 2011), using three iterations in the procedure to gain precision in the alignment. In our study, we performed a preliminary alignment of plant DGK against the *Saccharomyces cerevisiae* Dgk1p (renamed ScDGK1 in our analysis) sequence and demonstrated that the ATP-binding site is conserved in all DGK catalytic domains in plants. Conversely, the consensus sequence in ScDGK1 has been replaced by the sequence H_58_LKSHE_63_ in the catalytic CTP domain. This catalytic domain exhibits a much simpler and less varied amino-terminal regulatory domain than its ATP-dependent counterpart (Xie et al., 2015). Importantly, ScDGK1 is a unique CTP-dependent nuclear/endoplasmic reticulum membrane-associated enzyme that catalyzes the formation of PA from DAG in yeast (Fakas et al., 2011).

**Table 1:** List of diacylglycerol kinases (DGKs) found in different plant species.

Gene name	Gene locus	GenBank or UniProtKB accession	Length (aa)	Molecular mass (kDa)	cDNA/EST
*AtDGK1*	AT5G07920	NP_196409	728	80.0	BT004148
*AtDGK2*	AT5G63770	NP_201182	712	79.4	AY380783
*AtDGK3*	AT2G18730	NP_849980	488	53.9	AY141990
*AtDGK4*	AT5G57690	NP_200577	487	55.5	DQ447086
*AtDGK5a*	AT2G20900	AAM62810	491	55.3	AY085589
*AtDGK5b*	AT2G20900	NP_850007	509	57.4	AC006234
*AtDGK6*	AT4G28130	NP_194542	466	52.5	AL035524
*AtDGK7*	AT4G30340	NP_567845	492	54.6	AF360174
*BrDGK1*	XP_009125705	XP_009125705	724	79.2	XM_009127457
*BrDGK2*	XP_009150428	XP_009150428	714	79.3	XM_009152180
*BrDGK3*	XP_009102225	XP_009102225	482	53.1	XM_009103977
*BrDGK4*	XP_009132111	XP_009132111	482	53.6	XM_009133863
*CaDGK1*	XP_004508690	XP_004508690	731	81.3	XP_004508690
*CaDGK2*	XP_004503885	XP_004503885	705	78.7	XP_004503885
*CaDGK3*	XP_004507809	XP_004507809	482	53.3	XP_004507809
*CcDGK*	GSCOC_T00004730001	CDP18396	732	80.8	HG739316
*CmDGK*	XP_008448453	XP_008448453	729	80.7	XP_008448453
*CsDGK1a*	XP_011650101	XP_011650101	734	81.1	XP_011650101
*CsDGK1b*	XP_011650112	XP_011650112	731	81.1	CM002922
*FvDGK1*	XP_004299317	XP_004299317	726	80.2	XP_004299317
*FvDGK2*	XP_011471031	XP_011471031	708	78.7	XP_011471031
*GmDGK1*	GLYMA17G08510	XP_003549561	727	80.9	XM_003549513
*GmDGK2*	XP_006580353	XP_006580353	725	78.6	XP_006580353
*GmDGK3*	GLYMA06G30185	XP_014632628	480	53.7	XM_014777142
*HvDGK*	M0X7X8	M0X7X8	722	80.2	M0X7X8
*MaDGK*	XP_009404759	XP_009404759	727	78.2	XP_009404759
*MdDGK1*	MDP0000900186	KM099881	707	79.3	CN874967
*MdDGK2*	MDP0000246501	KM099882	489	54.6	CN890995
*MdDGK3*	MDP0000276007	EB177954	724	80.2	EB177954
*MdDGK4*	MDP0000139683	GO512216	502	54.5	GO512216
*MdDGK5*	MDP0000401076	KM099880	522	58.5	CN892391
*MdDGK6*	MDP0000237723	GO552958	488	54.5	GO552958
*MdDGK7*	MDP0000286961	KM099883	737	81.6	EB110199
*MdDGK8*	MDP0000171640	DR992213	538	60.2	DR992213
*MtDGK*	MTR_4g109390	XP_013457969	727	80.6	XM_013602515
*OsDGK1*	OS04G54200	EAZ32109	541	60.3	CM000141
*OsDGK2*	OS08G08110	NP_001061130	502	55.8	AP008214
*OsDGK3*	OS02G54650	NP_001048345	488	53.9	AP005535
*OsDGK4*	OS12G38780	NP_001067111	705	78.7	DP000011
*OsDGK5*	OS03G31180	ABF96709	616	68.1	DP000009
*OsDGK6*	OS08G15090	BAD05689	527	57.1	AP005495
*OsDGK7*	OS01G57420	EEE55501	499	55.9	CM000138
*OsDGK8*	OS12G12260	ABG21922	663	72.8	DP000011
*PpDGK*	PRUPE_ppa002021mg	XP_007210336	728	80.3	XM_007210274
*PvDGK*	PHAVU_003G185800g	XP_007155250	727	81.0	XM_007155188
*SbDGK*	SORBIDRAFT_01g032250	XP_002467682	716	80.2	XM_002467637
*SlDGK1a*	AF198259	AAG23129	489	54.5	AF198259
*SlDGK1b*	AF198258	AF198258.1	511	57.4	AF198258
*StDGK*	XP_006356748	XP_006356748	739	80.6	XM_006356686
*TaDGK*	A0A096UKE0	A0A096UKE0	721	80.1	A0A096UKE0
*TcDGK*	TCM_014170	XP_007037513	728	80.5	XM_007037451
*VvDGK1*	VIT_17s0000g06970	XP_002281347	731	81.0	XM_002281311
*VvDGK2*	VIT_07s0031g02840	XP_002272045	714	79.8	XM_002272009
*VvDGK3*	VIT_11s0052g01840	XP_002271984	512	56.8	XM_002271948
*ZmDGK1*	GRMZM2G076911_P01	NP_001106237	714	78.6	EF088691
*ZmDGK2*	GRMZM2G094452_P01	NP_001106236	500	55.6	EF088690
*ZmDGK3*	GRMZM2G106578_P01	ABO16345	495	55.2	EF088692

*Data were retrieved from NCBI and UniProtKB. ^∗^For each gene name, the scientific names of the corresponding plant species were considered in the beginning of our nomenclature: At, *Arabidopsis thaliana*; Br, *Brassica rapa*; Ca, *Cicer arietinum*; Cc, *Coffea canephora*; Cm, *Cucumis melo*; Cs, *Cucumis sativus*; Fv, *Fragaria vesca*; Gm, *Glycine max*; Hv, *Hordeum vulgare*; Ma, *Musa acuminata*; Md, *Malus domestica*; Mt, *Medicago truncatula*; Os, *Oryza sativa*; Pp, *Prunus persica*; Pv, *Phaseolus vulgaris*; Sl, *Solanum lycopersicum*; St, *Solanum tuberosum*; Sb, *Sorghum bicolor*; Ta, *Triticum aestivum*; Tc, *Theobroma cacao*; Vv, *Vitis vinifera*; Zm, Zea mays*.

**FIGURE 2:**
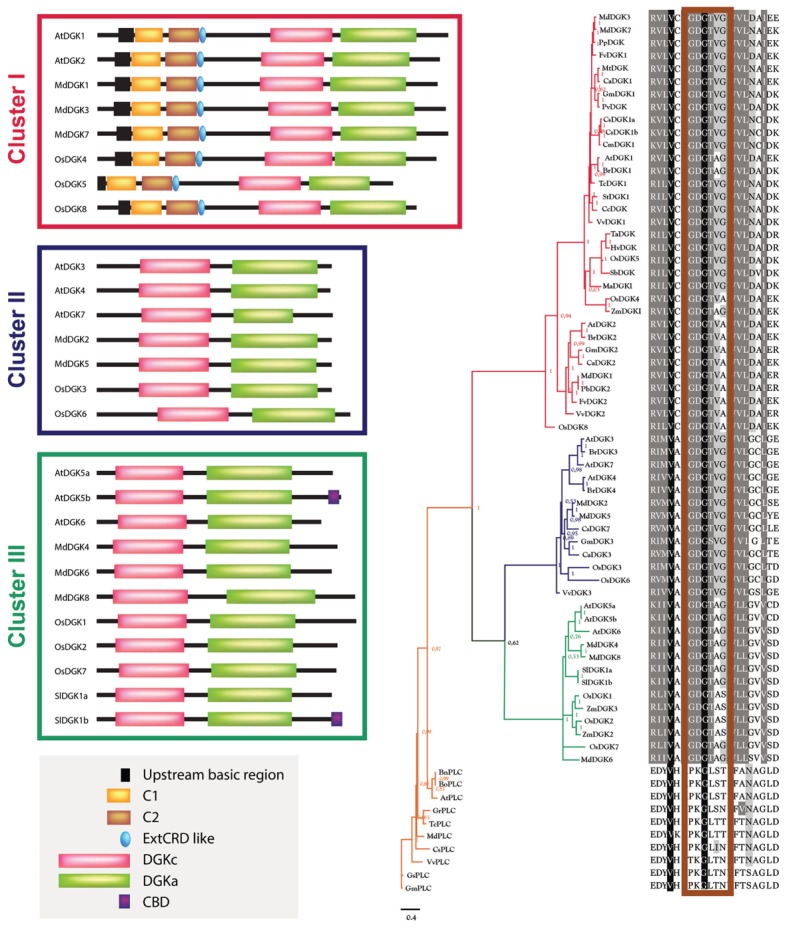
**Phylogenetic analysis of DGK and PLC enzymes from higher plants**. The phylogenetic analysis was built using the evolutive WAG method with variations described using a gamma-type distribution (rates = invgamma), default hyperparameters and three million generations (ngen = 3000000) in MrBayes3.2.5 software (Altekar et al., 2004). The subsequent probability values are indicated in the base of each clade. The sequence composition regarding the catalytic domain in each taxon in the multiple alignment is indicated in the right. The corresponding alignment was performed using Clustal-Omega1.2.1 software (Sievers et al., 2011), which uses HMM and a tree guide, constructed by the UPGMA method and a substitution matrix. This algorithm was iterated three times in order to avoid spurious results. The resulting clusters have been colored: red, Cluster I; blue, Cluster II; green, Cluster III; orange, External Group (PLCs). On the left of each DGK cluster, the domain distribution of some representative members is included and its structural composition is according to the studies carried out in tomato (Snedden and Blumwald, 2000), Arabidopsis (Gómez-Merino et al., 2004, 2005a; Vaultier et al., 2008; Arisz et al., 2009), rice (Ge et al., 2012), and apple (Li et al., 2015). The phylogenetic tree was visualized using the FigTree1.4.2 program (http://tree.bio.ed.ac.uk/). In the right of the tree, the ATP-binding site is indicated in the rectangle depicted in maroon over the alignment. The multiple sequence alignment result was visualized with GeneDoc 2.7 (Nicholas and Nicholas, 1997). The gray box in the bottom left corner includes the following domains: an upstream basic region; one or two DAG-binding domains (C1 and C2); an extended cysteine-rich domain (extCRD); a diacylglycerol kinase accessory domain (DGKa); a diacylglycerol kinase catalytic domain (DGKc); and calmodulin-binding domain (CBD).

The sequence alignment results were used as input for the construction of the phylogenetic tree, using MrBayes3.2.5 software (Altekar et al., 2004) to perform it. To have an evolutionary perspective of the DGK phylogeny, we added PLC proteins as an external group in our analysis (Supplementary Material 1). **Figure 2** shows the Bayesian phylogenetic tree of our results.

In **Figure 2**, the isoforms AtDGK1, AtDGK2, BrDGK1, BrDGK2, CaDGK1, CaDGK2, CcDGK, CsDGK1a, CsDGK1b, CmDGK1, FvDGK1, FvDGK2, GmDGK1, GmDGK2, HvDGK, MaDGK1, MdDGK3, MdDGK7, MtDGK, PvDGK, TcDGK1, StDGK1, TaDGK, OsDGK5, PpDGK, SbDGK, MdDGK1, OsDGK4, OsDGK8, PbDGK2, VvDGK1, VvDGK2, and ZmDGK1 encompass Cluster I; Cluster II consists of the isoforms AtDGK3, AtDGK4, AtDGK7, BrDGK3, BrDGK4, MdDGK2, MdDGK5, CsDGK7, GmDGK3, CaDGK3, OsDGK3, OsDGK6, and VvDGK3; and the isoforms AtDGK5a, AtDGK5b, AtDGK6, MdDGK4, MdDGK6, MdDGK8, SlDGK1a, SlDGK1b, OsDG1, OsDGK2, OsDGK7, ZmDGK2, and ZmDGK3 comprise Cluster III. The sequences BnPLC, BoPLC, AtPLC, GrPLC, TcPLC, MdPLC, CsPLC, VvPLC, GsPLC, and GmPLC belong to an external group of the phylogenetic tree. Considering this external PLC group, the first event of diversification occurred between DGKs of Cluster I and the rest of the sequences. After that, the second diversification event took place between proteins included in Clusters I and II.

**FIGURE 3:**
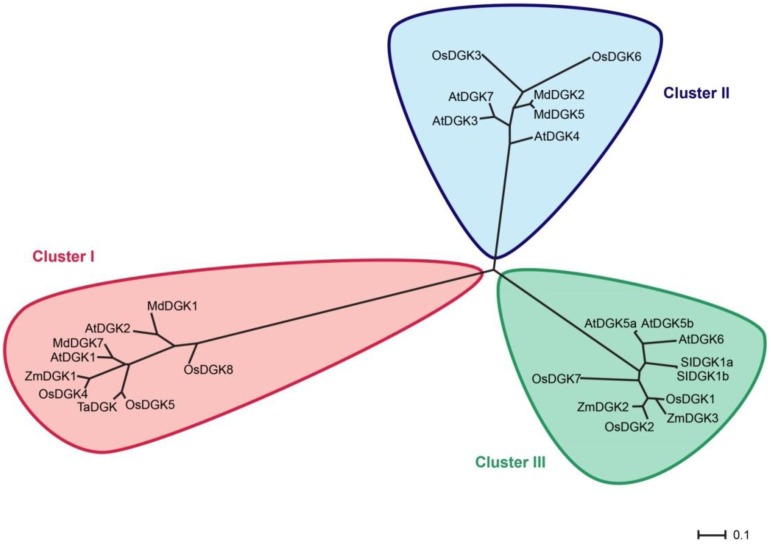
**Rooted phylogenetic tree of plant DGK isoforms**. For this analysis we used only DGK isoforms that had been reported in previous studies. In red, Cluster I; in blue, Cluster II; in green, Cluster III. The phylogenetic tree was visualized using the FigTree1.4.2 program (http://tree.bio.ed.ac.uk/). Protein names are according to **Table 1**.

In order to test whether our phylogenetic analysis was consistent with those previously reported, we constructed an additional rooted tree (**Figure 3**). Our results confirm that plant DGKs fall into three phylogenetic clusters, which is in full agreement with other studies reported by Gómez-Merino et al. (2004), Zhang et al. (2008), Ge et al. (2012), and Li et al. (2015). This second phylogenetic tree is also consistent with that presented in **Figure 2**.

### Expression Profiling Analysis of *DGK* Genes in Different Plant Tissues

**FIGURE 4:**
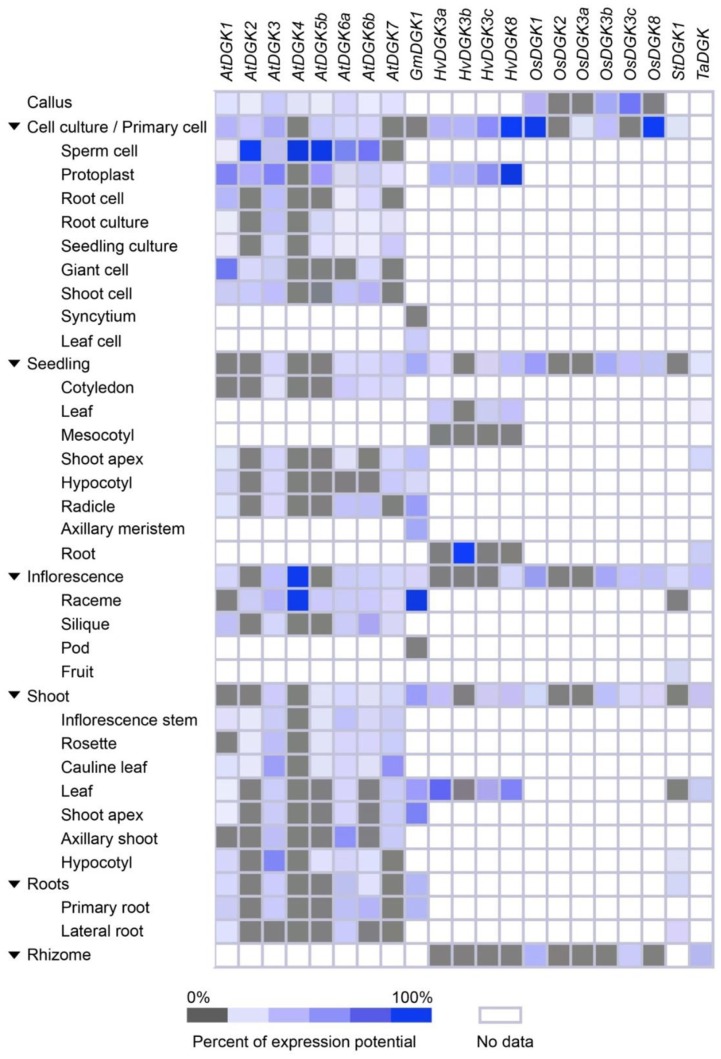
**Expression patterns of selected plant *DGK* genes, based on plant tissue types**. Tissue-specific expression in callus, cell culture, seedling, inflorescence, shoot, roots, and rhizome were retrieved from Genevestigator (https://genevestigator.com/gv/). Mean values were obtained from at least three replicates. Colors represent the intensity of expression (percentage of expression potential), from gray (0%) to dark blue (100%). Empty (colorless) boxes indicate that no data could be retrieved from the platform.

Genevestigator^6^ represents a high-performance bioinformatics search tool for gene expression analyses. It integrates a plethora of manually curated, well-described public experiments and accurately displays gene expression in response to diverse environmental contexts. We took advantage of this tool and analyzed transcriptional expression of *DGKs* genes in different tissues when available (**Figure 4**). Importantly, we were able to retrieve crucial expression data of *DGK* genes when Arabidopsis, barley, soybean, tomato, rice, or wheat plants were exposed to Ag, Al, As, Cd, Cr, Hg, or Na.

We found that *DGK* genes were overexpressed during cell culture, seedling, and inflorescent stages. At cell culture, induced expression of *AtDGK2*, *AtDGK4*, and *AtDGK5b* was observed in sperm cell; *HvDGK8* in protoplast; *OsDGK1* and *OsDGK8* in the whole cell culture and primary cell. In seedlings the gene *HvDGK3c* was found to be induced in roots. During inflorescence, the genes *AtDGK4* and *GmDGK1* were overexpressed in raceme. In general, all genes analyzed were expressed in all tissues, though at different levels. This is consistent with the expression data reported by Gómez-Merino et al. (2004), especially concerning *AtDGK2*, since such gene was found to be highly expressed in young tissues and flowers. In a study performed in reproductive organs of Arabidopsis, the published microarray data revealed that *AtDGK1*, *AtDGK3*, and *AtDGK5* are primarily expressed in pistils, stamens, and petals, while *AtDGK4* is highly expressed only in stamens. Instead, *AtDGK5* is slightly higher expressed in stamens and petals than in pistils (Yunus et al., 2015). Their qRT-PCR analyses showed that the expression of *AtDGK7* was the highest among DGK isoforms, while *AtDGK4* and *AtDGK6* were the lowest. Accordingly, Gómez-Merino et al. (2004) and Arana-Ceballos (2006) reported that the *AtDGK2* and *AtDGK7* genes, respectively, were indeed expressed in flower tissues. Moreover, six apple *DGK* genes (*DGK1*, *DGK2*, *DGK4*, *DGK5*, *DGK7*, and *DGK8*) were found to be highly expressed in stems and most of them in the flower as a whole (Li et al., 2015), which is consistent with the results retrieved from Genevestigator.

All together, these findings demonstrate that eight *DGK* gene probes from Arabidopsis, one from soybean, four from barley, six from rice, one from tomato, and one from wheat, displayed ubiquitous expression in most tissues analyzed. In general, all genes showed high expression in the young and reproductive tissues, pointing to a role of DGK and its enzymatic product, PA, in development and functions of floral organs.

### Expression Profiling Analysis of *DGK* Genes in Response to Beneficial Elements and Other Ions

**FIGURE 5:**
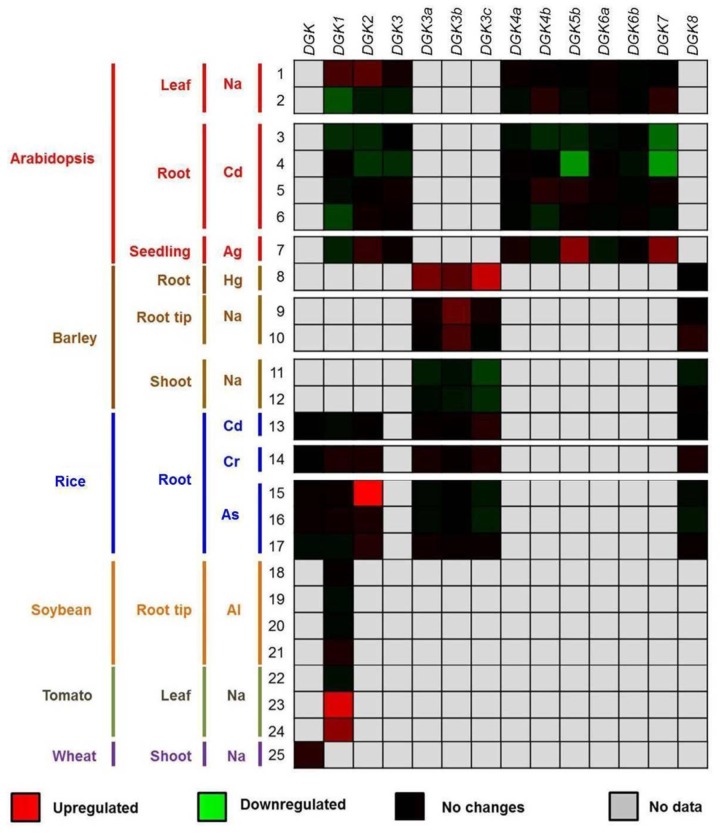
**Expression profiling data of *DGK* genes in response to beneficial elements, metal, and metalloid ions**. The up- or downregulation of the expression levels of *DGK* genes in the presence of Ag, Al, As, Cd, Cr, Hg, and Na are represented through red and green saturation colors, respectively. Experiment details are described in Supplementary Materials 2 and 3, as well as in Datasheet 1, while gene probe analyses are listed in Supplementary Material 4. Data were retrieved from the Genevestigator platform (https://genevestigator.com/gv/; Zimmermann et al., 2014). The experiments (1–25) were conducted in the presence of low concentrations of chemical elements. 1–7: Na (1–2), Cd (3–6), and Ag (7) in leaves (1–2), roots (3–6) and seedlings (7) of Arabidopsis; 8–12: Ag (7), Hg (8), and Na (9–12) in roots (8), root tips (9–10), and shoots (11–12) of barley; 13–17: Cd (13), Cr (14), and As (15–17) in rice roots; 18–21: Al in soybean root tips; 22–24: Na in tomato leaves; 25: Na in wheat shoots. In all cases, control experiments (where a determined element was not applied) were run in parallel.

We explored the gene expression profiling data deposited in the Genevestigator platform^6^ and found that various *DGK* genes are differentially regulated by Ag, Al, As, Cd, Cr, Hg, and Na (**Figure 5**).

Beneficial elements are not essential in most plant species. Instead, they may elicit hormetic responses by stimulatory effects when applied at low concentrations. Although up to now still largely unexplained, the plant growth enhancing effects of beneficial elements is gaining increased attention in relation to enhancing crop production (Belz and Cedergreen, 2010; Calabrese, 2014). In order to attain deeper knowledge on the effects of such elements on plant biology, we explored the transcript profiling data of *DGK* genes available in Genevestigator.

We have previously observed that *AtDGK2* transcript levels are induced in response to Al, one of the beneficial elements referred to in the literature (Blamey et al., 2015; Gómez-Merino et al., 2005b). Herein we report that some other plant *DGK* genes are indeed activated by beneficial elements and other ions (**Figure 5**). When barley plants were exposed to ∼1 μM Hg the expression of *HvDGK3a*, *HvDGK3b*, and *HvDGK3c* was significantly induced in root tissues. Similarly, in Arabidopsis roots the genes *AtDGK5b* and *AtDGK7* were highly transcribed in the presence of 10 μM Ag. In rice, plants exposed to 13.3 μM dibasic sodium arsenate (NaHAsO_4_) exhibited induced expression of *OsDGK2* in leaves, whereas in tomato the application of 200 mM Na enhanced the expression of *SlDGK1* also in leaves. Conversely, lower expression levels were detected in shoots, especially those of *AtDGK5b* and *AtDGK7* in response to 200 μM Cd.

## Discussion

Plants have evolved effective response strategies to react against environmental stimuli and protect themselves from various stress factors. One of the most important signaling pathways that mediate these responses is represented by the PPI, in which one of the emerging components is PA (Arisz et al., 2013; Saucedo-García et al., 2015). In the plasma membrane, PA can be produced principally by the PLD and PLC/DGK pathways. Interestingly, most of the PA synthetized in plants stimulated over a prolonged period is through the PLD pathway (Testerink and Munnik, 2011). Instead, a rapid PA accumulation in response to low temperature stress is generated through the PLC/DGK pathway (Arisz et al., 2013).

DGK activity has been reported in several plant species, including *Catharanthus roseus*, tobacco (*Nicotiana tabacum*), wheat, tomato, Arabidopsis, rice, and apple (Gómez-Merino et al., 2005a; Zhang et al., 2008; Li et al., 2015), and the molecular data bases reveal that they are present in a number of other crop plants such as maize, grape, sweet orange, and cotton. Nonetheless, detailed analysis of transcriptional activation of *DGK* genes and functional analysis of their corresponding protein products are still fragmentary. Two *DGK* cDNAs, *LeDGK1* and *LeCBDGK*, have been cloned from tomato and found to be derived from the same gene via alternative splicing. LeCBDGK, the protein product of *LeCBDGK*, contains a calmodulin (CaM)-binding domain (CBD). The two proteins lack the cysteine-rich domain (CRD) present in other eukaryotic DGKs, but are active *in vitro*. LeCBDGK (SlDGK1b in **Table 1**) is found both in associations with membranes and in soluble cell extracts. By contrast, LeDGK1 (SlDGK1a in **Table 1**) only associates with the membrane fraction, via a Ca^2+^/CaM-independent mechanism, which might represent a means of encoding specificity in cellular responses by alternative splicing (Snedden and Blumwald, 2000). In Arabidopsis, the *AtDGK1* cDNA has been isolated and reported to be mainly expressed in roots, shoots, and leaves, but its enzyme product was not active *in vitro* (Katagiri et al., 2001). However, Vaultier et al. (2008) found DGK activity in most membrane compartments, and speculated that AtDGK1 and AtDGK2 contribute to this activity. Two Arabidopsis *DGK* cDNAs (*AtDGK2* and *AtDGK7*) were cloned and their encoded enzymes were catalytically active in *in vitro* assays. *AtDGK2* transcripts are found in the whole plant except in stems and are induced by exposure to cold (4°C), pointing to a role in cold signal transduction (Gómez-Merino et al., 2004), whereas the *AtDGK7* gene is mainly found in flowers, young seedlings, and cauline leaves (Gómez-Merino et al., 2005a). In rice, it has been reported that the overexpression of the DGK gene *OsBIDK1* enhances disease resistance in transgenic tobacco (Zhang et al., 2008). Importantly, the inhibition of DGK activity drastically reduces root elongation and plant growth (Gómez-Merino et al., 2005a), indicating that DGKs may play a pivotal role not only in stress responses but also in developmental processes in plants. Whether this inhibition impact on plant development is related to an effect on the nutrient status of the plants remains to be elucidated. Nonetheless, with the highest DGK inhibitor dose used, plants exhibited a general chlorosis and died earlier as the inhibitor concentrations increased in the growth medium, which indeed points to a role of DGK enzymatic activity on nutrient status of the plant (Gómez-Merino et al., 2005a).

In terms of plant nutrition, essential elements are classified into macronutrients and micronutrients, according to the concentrations in which they are found in plant tissues. Apart from C, N, and O, the first group encompasses N, P, K, Ca, Mg, and S, which are found in plant tissues in concentrations of approximately 1000 mg kg^-1^ dry biomass weight. Instead, plant micronutrients, which are represented by Cl, Cu, Fe, Mg, Mo, Ni, and Zn, are found in concentrations close to 500 mg kg^-1^ of biomass in dry bases (Alcántar-González et al., 2016). Beneficial elements are not essential for plants, but when applied at low dosages, they may improve yield and quality parameters of some plant species of agricultural importance (Pilon-Smits et al., 2009; Trejo-Téllez et al., 2016). To date, the proposed beneficial elements include Al, Ce, Co, Fl, La, Na, Se, Si, Ti, V, and W (Pilon-Smits et al., 2009; Poschenrieder et al., 2013; Trejo-Téllez et al., 2016). Furthermore, other chemical elements such as Ag, As, Cd, and Hg may trigger hormetic effects in plants. Herewith we demonstrated that some *DGK* genes are induced in response to Ag, Al, Cd, Cr, Hg, and Na, suggesting a possible role of these genes and their protein products on plant nutrition.

We implemented an evolutive method in order to construct a consensus phylogenetic tree of DGKs from higher plants. In doing so, we took into consideration all DGK sequences in which enzymatic activity had been previously demonstrated, or that had been considered in other phylogenetic analyses. We found that plant DGK enzymes were distributed into three major clusters, as previously reported for Arabidopsis, rice, maize, wheat, tomato, and apple (Snedden and Blumwald, 2000; Gómez-Merino et al., 2004; Sui et al., 2008; Zhang et al., 2008; Li et al., 2015).

So far, all plant DGKs exhibit a bipartite catalytic region composed of a catalytic domain (DGKc; Pfam accession number PF00781) followed by an accessory domain (DGKa; Pfam accession number PF00609) at the C-terminus. Since DGKa is associated with DGKc, the former may contribute to the functionality of the catalytic domain. Whereas mammalian DGK enzymes can be classified into five groups according to sequence homology (Topham and Prescott, 2010), plant DGKs only form three phylogenetic clusters, and exhibit a simpler domain organization. Though less varied and complex, plant DGKs included in Cluster I resemble the closest sequence orthologous of metazoan enzymes with proteins that display one or two DAG-binding domains (C1), an upstream basic region and an extended CRD in their N-terminus (Gómez-Merino et al., 2004; Vaultier et al., 2008; Cacas et al., 2016). Conversely, Cluster II DGKs just harbor the DGKa and DGKc domains. Interestingly, Cluster III DGKs may exhibit a C-terminal CBD generated by alternative splicing (Arisz et al., 2009; Cacas et al., 2016).

The yeast *S. cerevisiae* was initially thought to lack a DGK, though Dgk1p, a novel type of DGK, utilizes CTP rather than the common ATP was discovered (Han et al., 2008a). This enzyme does not exhibit sequence similarity to DAG kinases from other species (Han et al., 2008b), and contains a short motif identified in a family of CTP-dependent phytol and dolichol kinases (Shridas and Waechter, 2006). Together with PA phosphatase, this enzyme controls the levels of PA and DAG for phospholipid synthesis, membrane growth, and lipid droplet formation (Qiu et al., 2013). The fact that the yeast DGK enzyme utilizes CTP, instead of ATP, as the phosphate donor in the reaction, explains why an ATP-dependent DAG kinase activity or a putative gene encoding a DAG kinase enzyme had not been identified in *S. cerevisiae* before (Han et al., 2008a).

Both experimental data previously reported and microarray analyses revealed that some *DGK* genes are expressed more prominently in young tissues, whereas their expression declines in older tissues. Importantly, most *DGK* transcripts are detectable in all tissues throughout all developmental stages, but their abundance decreases in tissues when plants become older.

Expression of *DGK* genes in response to both biotic and abiotic stimuli and stress factors has been proved (Gómez-Merino et al., 2004; Arisz et al., 2009; Pleskot et al., 2012; Ruelland et al., 2015). Accordingly, our survey revealed that some *DGK* genes are indeed regulated by Ag, Al, As, Cd, Cr, Hg, and Na, which may display hormetic dose–response curves.

According to Pilon-Smits et al. (2009), Ag can be considered as a potentially beneficial element, while information on the cellular basis for the positive effects of this element in plants is still fragmentary. Currently, silver nanoparticles (AgNPs) have remarkable uses in agricultural and environmental systems. Sharma et al. (2009) have pointed to a crucial role of AgNPs as antimicrobial agents. In plants, AgNPs impact oxidative stress-related gene expression, seed germination, and root elongation, leading to both positive and negative effects on plant growth (Cox et al., 2016). The final effects of AgNPs depend on nanoparticle (NP) size, shape, surface coating, and concentration used. Likewise, plant genotypes differ in their responses to NP exposure. In our survey, when Ag was applied to Arabidopsis plants, inducible gene expression was strongly evident for *AtDGK5b* and *AtDGK7.* Indeed, AgNPs have been implicated in signal transduction pathways involving kinases within the PI signaling pathway in eukaryotic cells (Kang et al., 2012), which suggests that Ag might influence other components of this pathway, including PLC and DGK in plants. This topic will be an interesting area for future study.

In the case of Al, Gómez-Merino et al. (2005b) reported induction of *AtDGK2* gene expression in hydroponically growing Arabidopsis plants exposed to 200 μM Al in the nutrient solution. In this study, Al induced the expression of the *DGK* gene rapidly, within 30 min after exposure, and it returned to the pretreated level 20 h after treatment (Gómez-Merino et al., 2005b). Likewise, Martínez-Estévez et al. (2001b) reported that Al entered coffee cells 30 min after treatment, and the signal was retained for up to 2 h. Furthermore, it was found that Al increases phosphorylation of particular proteins in cellular suspension cultures of coffee (Martínez-Estévez et al., 2001a). Subsequently, it was demonstrated that Al quickly (in 1 min) increased enzymatic activity of PLC as well as PI4K, PIP5K, and DGK (Martínez-Estévez et al., 2003), supporting the hypothesis that *DGK* genes might be implicated in the production of PA in response to Al. Nevertheless, this issue awaits further research at the molecular level. Just recently, Bojórquez-Quintal et al. (2014) reported that Al effects on primary root growth in coffee plants were dose-dependent: 100–300 μM Al stimulated primary root growth, while 500 μM induced damage to the root tips and inhibited primary root growth. Furthermore, 100 μM Al also increased the K and Ca contents by around 33 and 35% in roots, as compared to the control, which demonstrates the beneficial effects of Al when applied at low concentrations. Indeed, Al may improve nutrient status in the Al-resistant wheat cultivar Yecora, since this genotype retained larger concentrations of Ca^2+^ and Mg^2+^ in the leaves (Moustaka et al., 2016). Nonetheless, we have to keep in mind that Al exhibits hormetic effects in plants, which means that low dosages may induce beneficial responses in some genotypes, and higher concentrations are likely to cause toxicity in most plant species, interfering with cytoskeleton structure and function, disrupting Ca homeostasis, hampering P metabolism, and inducing oxidative stress (Blamey et al., 2015). In our survey, only the soybean *GmDGK1* gene was tested, showing no significant changes upon Al exposure. Whether other *DGK* genes are indeed induced by Al is still an open question that remains to be answered in future plant signaling studies.

Arsenic (As) is a metalloid occurring in natural environments in some abundance (1.2–1.4 ppm) in the Earth’s crust and in small quantities in rocks, soils, water bodies, and air. Hence, plants have evolved in the presence of this element, and it has been postulated that As might induce hormetic effects on plant growth. Although the mechanism of action is still unknown, it has been suggested that the growth benefit triggered by As arises from As stimulation of Pi uptake (Tu and Ma, 2003; Finnegan and Chen, 2012). However, because of its general toxic effects in living organisms, As is of considerable concern, and special care has to be taken in order to take advantage of its potential use in agriculture. In our study, As (in the form NaHAsO_4_) induced the expression of rice *OsDGK2*, but slightly downregulated that of *OsDGK3a* and *OsDGK8*. This fact, as well, points to a possible role of As in the PI signaling pathway.

In some plant species, Cd has been proved to trigger beneficial protective effects at low doses, in an hormetic dose–response manner (Liu et al., 2015). In fact, hormesis was found during the growth of switchgrass (*Panicum virgatum*) under hydroponic conditions, since plants were capable of developing better in the nutrient solution containing 100–175 μM Cd at pH 4.1–5.9, showing enlargement of the root length and surface, enhanced absorption of essential nutrients and increased biomass (Wang et al., 2015). Similarly, Liu et al. (2015) reported higher concentrations of chlorophylls (*a*, *b*, and total) and carotenoids, as well as relative water contents in leaves of *Lonicera japonica* plants receiving 0.5–5.0 mg L^-1^ Cd in the nutrient solution, as compared to the control. In our analysis, Cd repressed the transcriptional activity of *AtDGK1*, *AtDGK2*, *AtDGK3*, *AtDGK4b*, *AtDGK5b*, and *AtDGK7*. In rice, Cd slightly enhanced the expression of *OsDGK8*. It has been previously demonstrated that Cd affects the content of PPI in mammalian cells (Borikov and Kaliman, 1999). Just recently, Rajakumar et al. (2016) reported that Cd disrupts lipid metabolism in *S. cerevisiae*. As a hormetic factor, Cd is also a hazardous metal for plants when certain threshold concentrations in their cells are exceeded (Trejo-Téllez et al., 2014), as a result of its great toxicity inducing oxidative stress, genotoxicity, disruption of the photosynthetic apparatus, and inhibition of root metabolism (López-Millán et al., 2009; Han et al., 2012; Andresen and Küpper, 2013; Liu et al., 2015). Whether Cd interferes with the PI signaling pathway, and especially with the activity of PLC and DGK, is a question worthy of further study.

Cr has been generally referred to as an essential element for animals (Thorvaldsson and Jónsdóttir, 2005). In fact, Ma and Hooda (2010) established that trivalent chromium Cr(III) is an essential/beneficial nutrient that in trace amounts regulates the sugar and cholesterol metabolism in human and animal cells, though its hexavalent form Cr(VI) is a potent carcinogen and extremely toxic for those biological systems. Importantly, a recent report by the European Food Safety Association’s Panel on Dietetic Products, Nutrition and Allergies (EFSA NDA Panel, 2014) determined that there is no evidence of beneficial effects associated with Cr intake in human health, and setting of an adequate Cr intake level is also not appropriate. Concerning the plant system, though its abundance in the Earth’s crust ranges from 100 to 350 ppm (which is higher than that of essential elements like Ni, Zn, and Cu), Cr displays low solubility in soils and plants absorb just small amounts of this element. Hence, normal concentrations of Cr in plant tissues are between 0.02 and 1.0 mg kg^-1^, which depends on soil concentrations of this element and plant species (Ma and Hooda, 2010). Similar to humans and animals, Cr(VI) is much more toxic to plants than Cr(III). Just recently, Kabir (2016) reported that Cr stress tolerance in rice cv. Pokkali is not related to metal sequestration but is associated with reduced Fe transport and increased antioxidant defense. As an hormetic element, González et al. (2015) reported that low dosages of Cr (i.e., 2 and 4 mg L^-1^) enhanced defense response and maintained photosynthetic activity in *Eichhornia crassipes*, implying that the antioxidant defense system enzymes attempted to ensure the redox homeostasis. In our analysis, Cr slightly induced the expression of most *OsDGK* isoforms. Bovykin et al. (1999) found that Cr(III) ions are adsorbed on the bilayer lipid membrane surface, changing the intramembrane potential difference. As pH increases, the adsorption of those ions decreases. Furthermore, McCarty (2006) reported that bioactive Cr can induce the plasmalemmal Ca^2+^-ATPase in mammals, which is connected with the regulation of phospholipid levels (Govindaraju et al., 1989). In fact, it has been well documented that Cr alters phospholipid metabolism in animals (Tandon, 1982), but its implications on this pathway in plants is still at issue.

Low levels of Hg salts induce stress-dependent increase in glucose uptake in mammalian cells (Stearns, 2007). Furthermore, the antimicrobial properties of Hg are well documented (Lemire et al., 2013), though its use for agricultural proposes has been restricted since the 1990s (EPA, 2002). Hormetic effects of Hg have been proved in different organisms, including mammals (Schmidt et al., 2004), *Caenorhabditis elegans* (Helmcke and Aschner, 2010), *Anas platyrhynchos* (Heinz et al., 2012), and plants. In *Lemna minor* and *Allium cepa*, Subhadra et al. (1991) reported that low levels of aquatic Hg (0.001–1.0 mg L^-1^) accelerated catalase and peroxidase activities. In our survey, Hg application in barley resulted in a strong induction of all three *HvDGK3* genes (*a*, *b*, and *c*). Nevertheless, additional experimental evidence demonstrating the connection between Hg and the PI signaling pathway remains to be elucidated.

Na has been proved to be an essential element for halophytes (i.e., salt-tolerant plants), including C4 or CAM species that use phosphoenolpyruvate (PEP) to fix CO_2_ during photosynthesis, since Na mediates the regeneration of PEP from pyruvate. Additionally, Na has been widely reported to trigger hormetic responses on most plant taxa, including C3 plants (Maathuis, 2013). Importantly, in hydrated form, Na^+^ and K^+^ are chemically and structurally very similar. Consequently, in environments with low K^+^ conditions, Na^+^ can be useful for plants. Hence, several functions carried out by K^+^ plants, including some of the physiological and metabolic ones (i.e., osmotic regulation, guard cell movement, and cell expansion), can be fulfilled by Na^+^. Nevertheless, plant species significantly vary in their capacity to replace K^+^ with Na^+^ (Pilon-Smits et al., 2009). Na is also an excellent accompanying cation for long-distance transport (Subbarao et al., 2003). In our survey, we could observe that Na slightly induced the expression of *AtDGK1* and *AtDGK2* in shoots. On the other hand, the activity of both genes was slightly repressed in roots. In wheat, Na hardly induced the expression of *TaDGK* in shoots, while it differentially repressed its expression in roots, depending on the time of exposure and the dosage used. In barley, Na induced the expression of *HvDGK3b*, but repressed that of *HvDGK3c*. In tomato, Na strongly induced transcriptional activity of *SlDGK1*. Interestingly, Peters et al. (2002) reported that the non-specific phospholipase C5 (NPC5) and its derived lipid mediator DAG regulate lateral root development induced by sodium chloride (NaCl) in *Arabidopsis thaliana*. Moreover, another non-specific phospholipase C, NPC4, promotes responses to abscisic acid and tolerance to hyperosmotic stress induced by NaCl in Arabidopsis (Peters et al., 2010). These findings suggest a connection between Na and the PLC/DGK pathway in plants, which has to be further investigated.

Beneficial elements are emerging as potential biostimulators for agricultural proposes, since they can induce desirable plant responses in a hormetic manner. In light of the growing need for sustainable agriculture, the general global challenges related to climate change and an increasing population demanding more food, beneficial elements open up new possibilities to attain food security. Though cumulative evidence points to beneficial elements like Al and Na having a role in the regulation of *DGK* gene expression, the possible impact of dosages, chemical forms, time of application, and genotype responses remains to be further investigated. Additionally, understanding the roles of beneficial elements and other hormesis-inducing ions in different plant species under stress conditions may be a possible area of further study. Herewith we provide evidence for the first time that *DGK* genes encoding enzymes implicated in the synthesis of PA, an emerging second messenger, are responsive to Ag, Al, As, Cd, Cr, Hg, and Na. Importantly, we found most *DGK* genes ubiquitously expressed in plant tissues, and encoded proteins widely distributed in higher plants. PA, the protein product of DGK activity, has been proved to be a crucial second messenger. Its potential role as signaling mediator in response to beneficial elements and other ions could be of crucial significance for plants, providing the cell with spatial and transient information to perform better under limiting environment conditions, or simply to improve plant production and productivity. The discovery and description of the molecular bases of such relationships would be a noteworthy contribution to the field of agriculture.

## Author Contributions

HE-S and FG-M developed and designed the experiments and the survey strategies. LT-T supervised the physiological and plant nutrition experiments. PP-R and JH-C supervised the bioinformatics analyses. HE-S and FG-M wrote the manuscript. LT-T, PP-R, and JH-C revised the manuscript.

## Conflict of Interest Statement

The authors declare that the research was conducted in the absence of any commercial or financial relationships that could be construed as a potential conflict of interest.

## Footnotes

1 www.ncbi.nih.gov/blast

2 www.ncbi.nih.gov

3 http://www.uniprot.org/

4 http://mrbayes.sourceforge.net

5 https://genevestigator.com/gv/index.jsp

6 https://genevestigator.com/gv/
