# Supplementary material for: Diacylglycerol Kinases Are Widespread in Higher Plants and Display Inducible Gene Expression in Response to Beneficial Elements, Metal, and Metalloid Ions
Source: Front Plant Sci. 2017 Feb 7;8:129. doi: 10.3389/fpls.2017.00129 (PMC5293798; doi:10.3389/fpls.2017.00129)
Supplement: Supplementary file 1 [file Table_1.docx]

**Diacylglycerol kinases are widespread in higher plants and display inducible gene expression in response to beneficial elements, metal and metalloid ions**

Hugo F. Escobar-Sepúlveda, Libia I. Trejo-Téllez, Paulino Pérez-Rodríguez, Juan V. Hidalgo-Contreras and Fernando C. Gómez-Merino*

**Supplementary Material 1:** List of phospholipases C (PLC) enzymes selected as external group of the phylogenetic analysis. Data were retrieved from NCBI and UniProtKB.

| **Gene Name** | **Gene Locus** | **GenBank or UniProtKB Accession** | **Length (aa)** | **Molecular Mass (kDa)** | **cDNA/EST** |
| --- | --- | --- | --- | --- | --- |
| *AtPLC* | AT5G67130 | NP_569045 | 426 | 46.6 | AB020742 |
| *BnPLC* | BnaA07g12090D | XP_013653592 | 424 | 79.4 | LK031926 |
| *BoPLC* | XP_013599326 | XP_013599326 | 423 | 46.5 | XM_013743872.1 |
| *CsPLC* | Csa_2G279190 | XP_011649324 | 433 | 47.7 | CM002923 |
| *GmPLC* | GLYMA17G18740 | XP_006600946 | 432 | 47.1 | CM000850 |
| *GrPLC* | B456_003G025600 | XP_012469557 | 426 | 47.0 | CM001742 |
| *GsPLC* | glysoja_046316 | A0A0B2PWD0 | 431 | 47.0 | KN663493 |
| *MdPLC* | XP_008338332 | XP_008338332 | 424 | 45.7 | XM_008340110 |
| *TcPLC* | TCM_000654 | XP_007047314 | 425 | 46.6 | CM001879 |
| *VvPLC* | VIT_07s0031g00330 | XP_002282885 | 428 | 47.0 | FN595233 |

* For each gene name, the scientific names of the corresponding plant species were considered in the beginning of our nomenclature: At: *Arabidopsis thaliana*; Bn: *Brassica napus*; Bo: *Brassica oleracea*; Cs: *Cucumis sativus*; Gm: *Glycine max*; Gr: *Gossypium raimondii*; Gs: *Glycine soja*; Md: *Malus domestica*; Tc: *Theobroma cacao*; Vv: *Vitis vinifera*.
