# Supplementary material for: Diacylglycerol Kinases Are Widespread in Higher Plants and Display Inducible Gene Expression in Response to Beneficial Elements, Metal, and Metalloid Ions
Source: Front Plant Sci. 2017 Feb 7;8:129. doi: 10.3389/fpls.2017.00129 (PMC5293798; doi:10.3389/fpls.2017.00129)
Supplement: Supplementary file 2 [file Table_2.docx]

Diacylglycerol kinases are widespread in higher plants and display inducible gene expression in response to beneficial elements, metal and metalloid ions

Hugo F. Escobar-Sepúlveda, Libia I. Trejo-Téllez, Paulino Pérez-Rodríguez, Juan V. Hidalgo-Contreras and Fernando C. Gómez-Merino*

**Supplementary Material 2.** Summary of experiments testing the effect of beneficial elements, metal and metalloid ions on *DGK* gene expression. Gene expression levels can be visualized in **Figure 4**. Data was retrieved from the Genevestigator platform available at https://genevestigator.com/gv/ (Zimmermann et al., 2014).

| **N°** | **ID** | **Genotype tested** | **Experiment** | **Plant tissue** | **Developmen-tal Stage** |
| --- | --- | --- | --- | --- | --- |
| 1 | AT-00621 | Col-0 | 200 μM of Na_2_S for 10 days | Leaf | Developed flower |
| 2 | AT-00621 | Des1-1 | 200 μM of Na_2_S for 10 days | Leaf | Developed flower |
| 3 | AT-00464 | Col-0 | 50 μM of CdCl_2_ for 24 hours | Root | Young flower |
| 4 | AT-00551 | Col-0 | 200 μM of Cd^2+^ for 6 hours | Root | Young flower |
| 5 | AT-00464 | Oas-a1.1 | 50 μM of CdCl_2_ for 18 hours | Root | Young flower |
| 6 | AT-00464 | Oas-a1.1 | 50 μM of CdCl_2_ for 24 hours | Root | Young flower |
| 7 | AT-00113 | Col-0 | 10 μM of AgNO_3_ for 3 hours | Seedling | Seedling |
| 8 | HV-00036 | Graphic | 1 mM of HgCl_2_ | Root | Seedling growth |
| 9 | HV-00014 | Golden Promise | 150 mM of NaCl | Root tip | Seedling growth |
| 10 | HV-00014 | Maythorpe | 150 mM of NaCl | Root tip | Seedling growth |
| 11 | HV-00014 | Golden Promise | 150 mM of NaCl | Shoot | Seedling growth |
| 12 | HV-00014 | Maythorpe | 150 mM of NaCl | Shoot | Seedling growth |
| 13 | OS-00040 | IR64 | 100 μM of Cd for 24 hours | Root | Seedling |
| 14 | OS-00040 | IR64 | 100 μM of Cr for 24 hours | Root | Seedling |
| 15 | OS-00003 | Azucena | 13.3 μM of NaHAsO_4_ for 7 days | Root | Seedling |
| 16 | OS-00003 | Bala | 13.3 μM of NaHAsO_4_ for 7 days | Root | Seedling |
| 17 | OS-00040 | IR64 | 100 μM of NaHAsO_4_ for 24 hours | Root | Seedling |
| 18 | GM-00039 | P I 416937 | 10μM of Al^3+^ for 2 hours | Root tip | Germination |
| 19 | GM-00039 | P I 416937 | 10 μM of Al^3+^ for 12 hours | Root tip | Germination |
| 20 | GM-00039 | P I 416937 | 10 μM of Al^3+^ for 48 hours | Root tip | Main shoot growth |
| 21 | GM-00039 | P I 416937 | 10 μM of Al^3+^ for 72 hours | Root tip | Main shoot growth |
| 22 | LE-00028 | Ailsa Craig | 200 mM of NaCl for 24 hours | Leaf | Main shoot growth |
| 23 | LE-00003 | Money Maker | 200 mM of NaCl for 5 hours | Leaf | Main shoot growth |
| 24 | LE-00003 | PI365967 | 200 mM of NaCl for 5 hours | Leaf | Main shoot growth |
| 25 | TA-00021 | W4910 | 2.62 g/L of NaCl for 24 days. | Shoot | Tillering |

*ID: Genevestigator gene probes.
