# Supplementary material for: Diacylglycerol Kinases Are Widespread in Higher Plants and Display Inducible Gene Expression in Response to Beneficial Elements, Metal, and Metalloid Ions
Source: Front Plant Sci. 2017 Feb 7;8:129. doi: 10.3389/fpls.2017.00129 (PMC5293798; doi:10.3389/fpls.2017.00129)
Supplement: Supplementary file 3 [file Table_3.docx]

Diacylglycerol kinases are widespread in higher plants and display inducible gene expression in response to beneficial elements, metal and metalloid ions

Hugo F. Escobar-Sepúlveda, Libia I. Trejo-Téllez, Paulino Pérez-Rodríguez, Juan V. Hidalgo-Contreras and Fernando C. Gómez-Merino*

**Supplementary Material 3.** Genevestigator gene probes (ID) of *DGK* genes analyzed. Gene expression levels can be visualized in Figures 3 and 4. Data were retrieved from the Genevestigator platform available at https://genevestigator.com/gv/ (Zimmermann et al., 2014).

| **Gene Name** | **Genevestigator ID** |
| --- | --- |
| *AtDGK1* | 250556_at |
| *AtDGK2* | 247346_at |
| *AtDGK3* | 266062_at |
| *AtDGK4a* | 247873_at |
| *AtDGK4b* | 250816_at |
| *AtDGK5b* | 265385_at |
| *AtDGK6a* | 253852_at |
| *AtDGK6b* | 253853_at |
| *AtDGK7* | 253578_at |
| *GmDGK1* | GmaAffx.37977.1.A1_at |
| *HvDGK3a* | Contig1826_s_at |
| *HvDGK3b* | HY07L14u_at |
| *HvDGK3c* | Contig1829_at |
| *HvDGK8* | Contig18158_at |
| *OsDGK* | Os.52789.1.S1_at |
| *OsDGK1* | Os.53835.1.S1_at |
| *OsDGK2* | Os.52713.1.S1_at |
| *OsDGK3a* | Os.29043.1.S1_at |
| *OsDGK3b* | OsAffx.9692.1.S1_at |
| *OsDGK8* | Os.52901.1.S1_at |
| *OsDGK3c* | OsAffx.17375.1.S1_at |
| *SlDGK1* | Les.3526.1.S1_a_at |
| *TaDGK* | TaAffx.37747.1.S1_at |
