# Supplementary material for: Diacylglycerol Kinases Are Widespread in Higher Plants and Display Inducible Gene Expression in Response to Beneficial Elements, Metal, and Metalloid Ions
Source: Front Plant Sci. 2017 Feb 7;8:129. doi: 10.3389/fpls.2017.00129 (PMC5293798; doi:10.3389/fpls.2017.00129)
Supplement: Supplementary file 4 [file Table_4.docx]

Diacylglycerol kinases are widespread in higher plants and display inducible gene expression in response to beneficial elements, metal and metalloid ions

Hugo F. Escobar-Sepúlveda, Libia I. Trejo-Téllez, Paulino Pérez-Rodríguez, Juan V. Hidalgo-Contreras and Fernando C. Gómez-Merino*

**Supplementary Material 4.** Detailed description of experiments testing the effect of beneficial elements, metal and metalloid ions on *DGK* gene expression. Gene expression levels can be visualized in **Figure 4**. Experiment number (N°) is according to the Supplementary Material 2. Data were retrieved from the Genevestigator platform available at https://genevestigator.com/gv/ (Zimmermann et al., 2014).

| **N°** | **TREATMENTS** | **CONTROL** |
| --- | --- | --- |
| 1 | Rosette leaf samples of Col-0 grown for 20 days on soil, then irrigated with 200 µM Na_2_S for 10 days. Other growth conditions: 16 h light (120 μmol photons m^-2^ s^-1^) at 20°C / 8 h dark at 18°C. | Rosette leaf samples of Col-0 grown for 30 days on soil under 16 h light (120 μmol photons m^-2^ s^-1^) at 20°C / 8 h dark at 18°C cycles (irrigated with water). |
| 2 | Rosette leaf samples of des1-1 grown for 20 days on soil, then irrigated with 200 µM Na_2_S for 10 days. Other growth conditions: 16 h light (120 μmol photons m-^2^ s^-1^) at 20°C / 8 h dark at 18°C. | Rosette leaf samples of des1-1 grown for 30 days on soil under 16 h light (120 μmol photons m^-2^ s^-1^) at 20°C / 8 h dark at 18°C cycles (irrigated with water). |
| 3 | Shoot samples of Col-0 grown for 2 weeks on MS medium with 0.25% (w/v) Phytagel and 1% (w/v) sucrose, then transferred to hydroponic culture for 2 weeks, and then treated for 24 h with CdCl_2_ (50 μM final concentration). The hydroponics nutrient solution also contained 2 mM Ca(NO_3_)_2_, 0.5 mM KH_2_PO_4_, 0.75 mM MgSO_4_, 10 mM KNO_3_, 1.5 µM CuSO_4_, 2 µM ZnSO_4_, 10 µM MnSO_4_, 50µM H_3_BO_3_, 0.1 µM MoO_3_, 50 µM KCl, 50 µM Fe-Na-EDTA and was replaced once a week. Other growth conditions: 16 h white light (120 μmol photons m^-2^ s^-1^) at 20ºC / 8 h dark at 18ºC. | Shoot samples of Col-0 grown for 2 weeks on MS medium with 0.25% (w/v) Phytagel and 1% (w/v) sucrose, then transferred to hydroponic culture for 2 weeks. The hydroponics nutrient solution also contained 2 mM Ca(NO_3_)_2_, 0.5 mM KH_2_PO_4_, 0.75 mM MgSO_4_, 10 mM KNO_3_, 1.5 µM CuSO_4_, 2 µM ZnSO_4_, 10 µM MnSO_4_, 50 µM H_3_BO_3_, 0.1 µM MoO_3_, 50 µM KCl, 50 µM Fe-Na-EDTA and was replaced once a week. Other growth conditions: 16 h white light (120 μmol photons m^-2^ s^-1^) at 20ºC / 8 h dark at 18ºC. |
| 4 | Root samples of Col-0 grown hydroponically as described in Arteca and Arteca (2000): Physiol Plant. 108: 188-193; Gong et al. (2003): PNAS 100: 10118-10123, for 4 weeks under 16 h light / 8 h dark cycles at 28°C, then exposed for 6 h to 200 µM Cd^2+^, a non-essential toxic heavy metal. | Root samples of Col-0 grown hydroponically as described in Arteca and Arteca, 2000, Physiol Plant. 108: 188-193; Gong et al., 2003, PNAS 100: 10118-10123, for 4 weeks and 6 h under 16 h light / 8 h dark cycles at 28°C. |
| 5 | Root samples of oas-a1.1 mutant plants grown for 2 weeks on MS medium with 0.25% (w/v) Phytagel and 1% (w/v) sucrose, then transferred to hydroponic culture for 2 weeks, and then treated for 18 h with CdCl_2_ (50 μM final concentration). The hydroponics nutrient solution also contained 2 mM Ca(NO_3_)_2_, 0.5 mM KH_2_PO_4_, 0.75 mM MgSO_4_, 10 mM KNO_3_, 1.5 µM CuSO_4_, 2 µM ZnSO_4_, 10 µM MnSO_4_, 50 µM H_3_BO_3_, 0.1 µM MoO_3_, 50 µM KCl, 50 µM Fe-Na-EDTA and was replaced once a week. Other growth conditions: 16 h white light (120 μmol photons m^-2^ s^-1^) at 20ºC / 8 h dark at 18ºC. | Root samples of oas-a1.1 mutant plants grown for 2 weeks on MS medium with 0.25% (w/v) Phytagel) and 1% (w/v) sucrose, then transferred to hydroponic culture for 2 weeks. The hydroponics nutrient solution also contained 2 mM Ca(NO_3_)_2_, 0.5 mM KH_2_PO_4_, 0.75 mM MgSO_4_, 10 mM KNO_3_, 1.5 µM CuSO_4_, 2 µM ZnSO_4_, 10 µM MnSO_4_, 50 µM H_3_BO_3_, 0.1 µM MoO_3_, 50 µM KCl, 50 µM Fe-Na-EDTA and was replaced once a week. Other growth conditions: 16 h white light (120 μmol photons m^-2^ s^-1^) at 20ºC / 8 h dark at 18ºC. |
| 6 | Shoot samples of oas-a1.1 mutant plants grown for 2 weeks on MS medium with 0.25% (w/v) Phytagel and 1% (w/v) sucrose, then transferred to hydroponic culture for 2 weeks, and then treated for 24 h with CdCl_2_ (50 μM final concentration). The hydroponics nutrient solution also contained 2 mM Ca(NO_3_)_2_, 0.5 mM KH_2_PO_4_, 0.75 mM MgSO_4_, 10 mM KNO_3_, 1.5 µM CuSO_4_, 2 µM ZnSO_4_, 10 µM MnSO_4_, 50 µM H_3_BO_3_, 0.1 µM MoO_3_, 50 µM KCl, 50 µM Fe-Na-EDTA and was replaced once a week. Other growth conditions: 16 h white light (120 μmol photons m^-2^ s^-1^) at 20ºC / 8 h dark at 18ºC. | Shoot samples of oas-a1.1 mutant plants grown for 2 weeks on MS medium with 0.25% (w/v) Phytagel and 1% (w/v) sucrose, then transferred to hydroponic culture for 2 weeks. The hydroponics nutrient solution also contained 2 mM Ca(NO_3_)_2_, 0.5 mM KH_2_PO_4_, 0.75 mM MgSO_4_, 10 mM KNO_3_, 1.5 µM CuSO_4_, 2 µM ZnSO_4_, 10 µM MnSO_4_, 50 µM H_3_BO3, 0.1 µM MoO_3_, 50 µM KCl, 50 µM Fe-Na-EDTA and was replaced once a week. Other growth conditions: 16 h white light (120 μmol photons m^-2^ s^-1^) at 20ºC / 8 h dark at 18ºC. |
| 7 | AgNO_3_ (ethylene inhibitor); 10 µM AgNO_3_ for 3 h. | Mock treatment for 3 h. |
| 8 | Root samples of cultivar Graphic grown on sand in 1 L pots for 15 days and irrigated with the half strength Hoagland's solution containing 1 mM HgCl_2_. Other growth conditions: glasshouse, 16°C, 45% relative humidity, maximum irradiance: 378 W m^-2^. | Root samples of cultivar Graphic grown on sand in 1l pots for 15 days and irrigated with the half strength Hoagland's solution. Other growth conditions: glasshouse, 16°C, 45% relative humidity, maximum irradiance: 378 W m^-2^. |
| 9 | On day 16 after germination (3-4 leaf stage), a salinity stress of was imposed on Golden Promise seedlings over a period of five days in five equal steps to reach a final concentration of 17 dS m^-1^ (~150 mM NaCl). CaCl_2_ was added with NaCl to maintain a 10:1 molar ratio of Na^+^: Ca^2+^. The system was allowed to stabilize for five days. On day 25 (5-6 leaf stage) root (2 cm of the root tips) tissue was harvested and snap frozen for RNA extraction. | Root (2 cm of the root tips) tissue of age-matched untreated Golden Promise seedlings. |
| 10 | On day 16 after germination (3-4 leaf stage), a salinity stress of was imposed on Maythorpe seedlings over a period of five days in five equal steps to reach a final concentration of 17 dS m^-1^ (~150 mM NaCl). CaCl_2_ was added with NaCl to maintain a 10:1 molar ratio of Na^+^: Ca^2+^. The system was allowed to stabilize for five days. On day 25 (5-6 leaf stage) root (2 cm of the root tips) tissue was harvested and snap frozen for RNA extraction. | Root (2 cm of the root tips) tissue of age-matched untreated Maythorpe seedlings. |
| 11 | On day 16 after germination (3-4 leaf stage), a salinity stress of was imposed on Golden Promise seedlings over a period of five days in five equal steps to reach a final concentration of 17 dS m^-1^ (~150 mM NaCl). CaCl_2_ was added with NaCl to maintain a 10:1 molar ratio of Na^+^: Ca^2+^. The Shoot (crown and growing point) tissue of age-matched untreated Golden Promise seedlings system was allowed to stabilize for five days. On day 25 (5-6 leaf stage) "shoot" (crown and growing point) tissue was harvested and snap frozen for RNA extraction. | Shoot (crown and growing point) tissue of age-matched untreated Golden Promise seedlings. |
| 12 | On day 16 after germination (3-4 leaf stage), a salinity stress of was imposed on Maythorpe seedlings over a period of five days in five equal steps to reach a final concentration of 17 dS m^-1^ (~150 mM NaCl). CaCl_2_ was added with NaCl to maintain a 10:1 molar ratio of Na^+^: Ca^2+^. The system was allowed to stabilize for five days. On day 25 (5-6 leaf stage) "shoot" (crown and growing point) tissue was harvested and snap frozen for RNA extraction. | Shoot (crown and growing point) tissue of age-matched untreated Maythorpe seedlings. |
| 13 | Root samples of cultivar IR64 germinated for 5 days at 37°C, then grown in Hewitt solution for 10 days, and then treated with 100 µM cadmium (Cd, CdCl_2_) for 24 h. Other growth conditions: 16 h light (115 μmol m^-2^ s^-1^) / 8 h dark; 25 ± 2°C. | Root samples of cultivar IR64 germinated for 5 days at 37°C, then grown in Hewitt solution for 11 days under 16 h light (115 μmol m^-2^ s^-1^) / 8 h dark cycles at 25 ± 2°C. |
| 14 | Root samples of cultivar IR64 germinated for 5 days at 37°C, then grown in Hewitt solution for 10 days, and then treated with 100 µM chromium VI (Cr(VI), K_2_Cr_2_O_7_) for 24 h. Other growth conditions: 16 h light (115 μmol m^-2^ s^-1^) / 8 h dark; 25 ± 2°C. Cr(VI) at the concentration of 100 µM inhibits shoot and root growth (Dubey et al., 2010, BMC Genomics. 11: 648). | Root samples of cultivar IR64 germinated for 5 days at 37°C, then grown in Hewitt solution for 11 days under 16 h light (115 μmol m^-2^ s^-1^) / 8 h dark cycles at 25 ± 2°C. |
| 15 | Root samples of rice variety Azucena grown for 7 days in hydroponics on phosphate-free nutrient solution containing 0.1 mM Mg^2+^ and SO_4_^2-^, 0.2 mM Ca^2+^ and K^+^, 0.6 mM NO3-, and 1 ppm (13.3 µM) arsenate (Na_2_HAsO_4_). | Root samples of rice variety Azucena grown for 7 days in hydroponics on phosphate-free nutrient solution containing 0.1 mM Mg^2+^ and SO_4_^2-^, 0.2 mM Ca^2+^ and K^+^, and 0.6 mM NO_3_^-^. |
| 16 | Root samples of rice variety Bala grown for 7 days in hydroponics on phosphate-free nutrient solution containing 0.1 mM Mg^2+^ and SO_4_^2-^, 0.2 mM Ca^2+^ and K^+^, 0.6 mM NO3-, and 1 ppm (13.3 µM) arsenate (Na_2_HAsO_4_). | Root samples of rice variety Bala grown for 7 days in hydroponics on phosphate-free nutrient solution containing 0.1 mM Mg^2+^ and SO_4_^2-^, 0.2 mM Ca^2+^ and K^+^, and 0.6 mM NO_3_^-^. |
| 17 | Root samples of cultivar IR64 germinated for 5 days at 37°C, then grown in Hewitt solution for 10 days, and then treated with 100 µM arsenate (Na_2_HAsO_4_) for 24 h. Other growth conditions: 16 h light (115 μmol m^-2^ s^-1^) / 8 h dark; 25 ± 2°C. | Root samples of cultivar IR64 germinated for 5 days at 37°C, then grown in Hewitt solution for 11 days under 16 h light (115 μmol m^-2^ s^-1^) / 8 h dark cycles at 25 ± 2°C. |
| 18 | Primary root tip (1 cm) samples of PI 416937 line germinated at 25°C for 3 days on germination paper moistened with deionized water, then incubated in a solution (pH 4.3) containing 10 μM aluminium (Al^3+^) and 800 μM CaCl_2_ for 2 h (growth chamber set to 16 h light (100 μmol photons m^-2^ s^-1^) at 28°C / 8 h dark at 20°C). | Primary root tip (1cm) samples of PI 416937 line germinated at 25°C for 3 days on germination paper moistened with deionized water, then incubated in a solution (pH 4.3) containing 800 μM CaCl_2_ for 2 h (growth chamber set to 16 h light (100 μmol photons m^-2^ s^-1^) at 28°C / 8 h dark at 20°C). |
| 19 | Primary root tip (1cm) samples of PI 416937 line germinated at 25°C for 3 days on germination paper moistened with deionized water, then incubated in a solution (pH 4.3) containing 10 μM aluminium (Al^3+^) and 800 μM CaCl_2_ for 12 h (growth chamber set to 16 h light (100 μmol photons m^-2^ s^-1^) at 28°C / 8 h dark at 20°C). | Primary root tip (1cm) samples of PI 416937 line germinated at 25°C for 3 days on germination paper moistened with deionized water, then incubated in a solution (pH 4.3) containing 800 μM CaCl_2_ for 12 h (growth chamber set to 16 h light (100 μmol photons m-2 s^-1^) at 28°C / 8 h dark at 20°C). |
| 20 | Primary root tip (1 cm) samples of PI 416937 line germinated at 25°C for 3 days on germination paper moistened with deionized water, then incubated in a solution (pH 4.3) containing 10 μM aluminium (Al^3+^) and 800 μM CaCl_2_ for 48 h (growth chamber, 16 h light (100 μmol photons m^-2^ s^-1^) at 28°C / 8 h dark at 20°C). | Primary root tip (1cm) samples of PI 416937 line germinated at 25°C for 3 days on germination paper moistened with deionized water, then incubated in a solution (pH 4.3) containing 800 μM CaCl_2_ for 48 h (growth chamber, 16 h light (100 μmol photons m^-2^ s^-1^) at 28°C / 8 h dark at 20°C). |
| 21 | Primary root tip (1cm) samples of PI 416937 line germinated at 25°C for 3 days on germination paper moistened with deionized water, then incubated in a solution (pH 4.3) containing 10 μM aluminium (Al^3+^) and 800 μM CaCl_2_ for 72 h (growth chamber, 16 h light (100 μmol photons m^-2^ s^-1^) at 28°C / 8 h dark at 20°C). | Primary root tip (1cm) samples of PI 416937 line germinated at 25°C for 3 days on germination paper moistened with deionized water, then incubated in a solution (pH 4.3) containing 800 μM CaCl_2_ for 72 h (growth chamber, 16 h light (100 μmol photons m^-2^ s^-1^) at 28°C / 8 h dark at 20°C). |
| 22 | 3rd leaf samples of cultivar Ailsa Craig grown for 4 weeks in a glasshouse on soil in 2 L pots, then sprayed with distilled H_2_O until run-off, incubated in the glasshouse for 24 h, then watered with 100 mM NaCl via pot-watering till maximum soil saturation was reached, and kept for 24 h in the glasshouse. Other plant growth conditions: 14 h light at 22°C / 10 h dark at 18°C cycles. | 3rd leaf samples of cultivar Ailsa Craig grown for 4 weeks in a glasshouse on soil in 2L pots, then sprayed with distilled H_2_O until run-off, incubated in the glasshouse for 24 h, then watered with H_2_O via pot-watering till maximum soil saturation was reached, and kept for 24 h in the glasshouse. Other plant growth conditions: 14 h light at 22°C / 10 h dark at 18°C cycles. |
| 23 | Whole plant samples of cultivar MoneyMaker (developmental stage: six leaves visible) treated with 1/4 strength Hoagland solution containing 200 mM NaCl for 5 h in aerated hydroponic tanks. Growth conditions prior the treatment: sterilized seeds were germinated in distilled water for one week at 26°C,16 h light (1500 lux) / 8 h dark, seedlings were then grown for 3 weeks on sterile sand supplemented with 1/4 strength Hoagland solution and finally transferred to hydroponic tanks with 1/4 strength Hoagland solution for 3 days. | Untreated whole plant samples of cultivar MoneyMaker (developmental stage: six leaves visible). Growth conditions: sterilized seeds were germinated in distilled water for one week at 26°C,16 h light (1500 lux) / 8 h dark, seedlings were then grown for 3 weeks on sterile sand supplemented with 1/4 strength Hoagland solution and finally transferred to hydroponic tanks with 1/4 strength Hoagland solution for 3 days and 5 h. |
| 24 | Whole plant samples of wild tomato, *Solanum pimpinellifolium*, line PI365967 (developmental stage: six leaves visible) treated with 1/4 strength Hoagland solution containing 200 mM NaCl for 5 h in aerated hydroponic tanks. Growth conditions prior the treatment: sterilized seeds were germinated in distilled water for one week at 26°C,16 h light (1500 lux) / 8 h dark, seedlings were then grown for 3 weeks on sterile sand supplemented with 1/4 strength Hoagland solution and finally transferred to hydroponic tanks with 1/4 strength Hoagland solution for 3days. | Untreated whole plant samples of wild tomato, *Solanum pimpinellifolium*, line PI365967 (developmental stage: six leaves visible). Growth conditions: sterilized seeds were germinated in distilled water for one week at 26°C,16 h light (1500 lux) / 8 h dark, seedlings were then grown for 3 weeks on sterile sand supplemented with 1/4 strength Hoagland solution and finally transferred to hydroponic tanks with 1/4 strength Hoagland solution for 3 days and 5 h. |
| 25 | Shoot samples of W4910 line grown for 42 days in a greenhouse (mid-March through mid-April, natural light, diurnal temperature range 16°C - 29°C, the USDA-ARS Forage and Range Research Laboratory, Logan, Utah, USA) and treated with increasing concentrations of salt beginning 3 days after planting in silica sand (plants were submerged twice weekly in salt containing complete nutrient mix starting with the initial electrical conductivity (EC) of 6 dS/m (0.92 g/L NaCl, 2.25 g/L CaCl_2_·H_2_O) and increasing EC weekly by an increment of 6 to a final EC of 30 dS/m (2.62 g/L NaCl, 14.74 g/L CaCl_2_·H_2_O). | Shoot samples of W4910 line grown for 42 days in a greenhouse (mid-March through mid-April, natural light, diurnal temperature range 16°C - 29°C, the USDA-ARS Forage and Range Research Laboratory, Logan, Utah, USA) in silica sand. Beginning 3 days after planting, plants were submerged twice weekly in complete nutrient mix. |
